# KRAS and VEGF gene 3'-UTR single nucleotide polymorphisms predicted susceptibility in colorectal cancer

**DOI:** 10.1371/journal.pone.0174140

**Published:** 2017-03-22

**Authors:** Minnan Yang, Xiuli Xiao, Xiaorui Xing, Xin Li, Tian Xia, Hanan Long

**Affiliations:** 1 College of Preclinical Medicine, Southwest Medical University, Luzhou, Sichuan, China; 2 Department of Pathology, the Affiliated Hospital of Southwest Medical University, Luzhou, Sichuan, China; 3 Department of Gastrointestinal surgery, Chengdu University of Traditional Chinese Medicine Affiliated Hospital, Chengdu, Sichuan, China; 4 Department of Science and Technology, Southwest Medical University, Luzhou, Sichuan, China; National Cancer Center, JAPAN

## Abstract

Single nucleotide polymorphisms (SNPs) in tumor-related genes have been reported to play important roles in cancer development. Recent studies have shown that 3’-untranslated regions (UTR) polymorphisms are associated with the occurrence and prognosis of cancers. The aim of this study is to analyze the association between KRAS and VEGF gene 3’-UTR SNPs and genetic susceptibility to colorectal cancer (CRC). In this case-control study of 371 CRC cases and 246 healthy controls, we analyzed the association between one SNP (rs1137188G > A) in the KRAS gene and four SNPs (rs3025039C > T, rs3025040C > T, rs3025053G > A and rs10434A > G) in the VEGF gene and CRC susceptibility by the improved multiplex ligase detection reaction (iMLDR) method. We checked the selected SNPs’ minor allele frequency and its distribution in the frequency of Chinese people by Hap-map database and Hardy-Weinberg equilibrium, and used multivariate logistic regression models to estimate adjusted odds ratios (AORs) and 95% confidence intervals (95% CIs). We found that the rs3025039C variant genotype in the VEGF gene was associated with a significant protection for CRC (AOR = 0.693, 95% CI = 0.485–0.989; *P* = 0.043 for CC and CT+TT). Nevertheless, the difference was no longer significant after Bonferroni correction (Bonferroni-adjusted *P* = 0.172). In genetic polymorphisms analysis, we found that the KRAS rs1137188 variant AA genotype had higher portion of tumor size (≥ 5 cm) (*P =* 0.01; Bonferroni-adjusted *P =* 0.04), which suggested that the rs1137188 variant AA genotype may significantly be associated with increased progression of CRC. In conclusion, our study suggested that these five SNPs in the KRAS gene and the VEGF gene were not associated with CRC susceptibility in Han Chinese in Sichuan province.

## Introduction

Colorectal cancer (CRC) is the third most common cancer, the fourth leading cause of cancer-related death globally, and the second most common cancer in terms of the number of individuals living with cancer for five years or more, making up about 10% of all malignant diagnoses. It is been estimated that 1.4 million people are diagnosed with CRC per year, 65% of whom are found in developed countries; approximately 700,000 die of this disease per year; and 3.5 million individuals are living with CRC [1]. CRC is a complex disease that is influenced by multiple factors [2–4], however, numerous evidences have suggested that environmental factors and genetic variations are postulated to significantly affect the risk of CRC [5, 6]. In addition, single nucleotide polymorphisms (SNPs) also play an important role in the development and progression of CRC [7–9]. At present, sequencing of the human genome has revealed about 18.7 million SNPs that are thought to account for 90% of all sequence variation [10].

The KRAS proto-oncogene belongs to the RAS GTPase family which regulates cellular proliferation, apoptosis and other important biological processes [11]. KRAS protein plays a key role in the EGFR signaling pathway and oncogenic mutations in protein can drive downstream activation of this pathway even in the absence of upstream EGFR activation. The KRAS mutation is an essential step which is thought to contribute to cancer development by driving proliferation of cells and resisting to apoptosis with initiated mutations. KRAS 3’-untranslated regions (UTR) of human contains multiple putative tumor suppressor lethal-7 (let-7) complementary sites (LCS). The SNPs of KRAS 3’-UTR may prevent the let-7 miRNA from binding to KRAS and regulate the activity of KRAS so as to adjust the expression of its protein. Previous studies have investigated that the SNPs in the KRAS 3’-UTR might cause high levels of the KRAS oncogenic protein and lower levels of the let-7 miRNA, the overexpressed KRAS oncogenic protein can increase the activation of the RAF/MEK/MAPK pathway, which might promote the tumorigenesis of CRC [12, 13].

The human vascular endothelial growth factor (VEGF) gene is located on chromosome 6 at location 6p21.3 and consists of 8 exons [14]. VEGF gene is highly polymorphic, and its promoter, 5’-, and 3’-UTR has a variety of SNPs [15]. VEGF SNPs in the 3’-UTR have been found to be associated with variations in VEGF protein production [16]. These SNPs could result in high expression of the VEGF gene and increase tumor-related angiogenesis and metastasis, playing a key role in a series of pathologic processes involved in tumor growth and metastasis [17]. Moreover, VEGF-involved angiogenesis pathways are also believed to be an important target of chemotherapeutic treatment in CRC. VEGF mRNA expression influences tumor tissues by the combination of 3’-UTR polymorphic alleles present. Studies showed that polymorphic events affecting the mRNA expression of VEGF gene could affect the survival duration of cancer patients having received anti-angiogenic treatment [18]. Therefore, VEGF gene polymorphisms might also serve as potential molecular biomarkers to predict clinical outcomes [14].

Genetic variations in the KRAS gene and VEGF gene have been reported to influence protein translation efficiency and modulate gene expression. They have also been reported to associate with the clinical outcome of CRC [19–21]. However, although these studies have shown 90% of KRAS mutations occur in codon 12 or 13, there are still a lot of mutations that occur in the 3’-UTR. Moreover, no study has been reported on the association between the genetic variations of VEGF gene 3’-UTR and the risk of CRC in the Chinese population. In the present study, we analyzed the association of KRAS with VEGF gene 3’-UTR SNPs and genetic susceptibility to CRC among Chinese to determine the genetic effects of Chinese in relation to CRC.

## Materials and methods

### Ethics statement

The procedure of the study was approved by the Ethics Committee of Southwest Medical University and the Medical Ethics Committee of the Affiliated Hospital of Southwest Medical University (No. 2012-00-72; 2012-01-72). Each participant provided a written informed consent with a signature.

### Subjects

All subjects were collected from the Department of Oncology, the Affiliated Hospital of Southwest Medical University between January 2012 and June 2014. A total of 617 subjects were involved in the current study. The case group comprised of 371 patients with histopathologically confirmed CRC. Patients were included without restrictions on age, gender, ethnicity, or clinical stage. Details of demographic characteristics and clinical data of the subjects were collected by medical record review, including age, gender, smoking status, alcohol consumption, body mass index (BMI), tumor site, recurrence/metastasis status, and Dukes stage. Patients with genetic relationship, recurring CRC, history of cancer, tumor chemotherapy, radiotherapy, or no detailed personal information and clinicopathological data were excluded from this study. The control group comprised of 246 age-matched healthy volunteers who were randomly selected from 1792 individuals during the same time period when the cases were recruited. For this study, smoking status was classified as smokers and non-smokers. The subjects who smoked at the time and/or had smoked at least 100 cigarettes during their lifetime were classified as smokers and those who consumed 100 ml liquor in 30 days were classified as alcohol consumers. All participants donated their blood sample for genomic DNA extraction in this study. All subjects were Han Chinese (Sichuan province).

### DNA isolation

EDTA-anti-coagulated venous blood samples were preserved at -70°C. DNA was obtained from leukocytes of each blood sample using a TIANamp Blood DNA Kit (TIANGEN Biotech, Beijing, China) according to the manufacturer’s instructions. Concentration of DNA was measured by using Qubit Fluorometer (Invitrogen/Life Technologies, Carlsbad, CA).

### SNP selection and genotyping

The potentially functional SNPs of KRAS and VEGF gene in 3’-UTR were selected from NCBI dbSNP database (http://www.ncbi.nlm.nih.gov/projects/SNP/). Meanwhile, we selected SNPs with minor allele frequency (MAF) > 5% from Asian population in the HapMap database (http://hapmap.ncbi.nlm.nih.gov/). Eventually, one SNP in the KRAS 3’-UTR (rs1137188G > A) and four SNPs in the VEGF 3’-UTR (rs3025039C > T, rs3025040C > T, rs3025053G > A and rs10434A > G) were chosen and included in the analysis. All the five SNPs were genotyped by Shanghai Genesky Bio-Tech Co., Ltd. (http://biotech.geneskies.com/en/index.php/Index/fuwuer/id/28.html) using the improved multiplex ligase detection reaction (iMLDR) method, the primers are listed in Table 1. To verify the reproducibility, the assays were repeated for 5% of the samples at random as a quality control for genotyping, and the quality control samples confirmed 100% concordance.

**Table 1:** SNPs and PCR primer for KRAS and VEGF allele genotyping.

Gene	SNPs	Chromosome position	PCR primer
**KRAS**	rs1137188	25206418	rs1137188F: TAGAGCCTAGAATGCCTACTTGGGAAC
			rs1137188R: GATGTAGATGGGCATTTTTTTAAGGTAGTG
**VEGF**	rs3025039	43784799	rs3025039F: AAGGAGCCTCCCTCAGGGTTTC
			rs3025039R: tGTGGGTGGGTGTGTCTACAGG
	rs3025040	43785314	rs3025040F: TCTACCCCAGGTCAGACGGACA
			rs3025040R: CAGGTCTCCTGGGGGGACAG
	rs3025053	43785588	rs3025053F: TCTACCCCAGGTCAGACGGACA
			rs3025053R: CAGGTCTCCTGGGGGGACAG
	rs10434	43785475	rs10434F: TCTACCCCAGGTCAGACGGACA
			rs10434R: CAGGTCTCCTGGGGGGACAG

SNP: Single nucleotide polymorphisms; PCR: polymerase chain reaction; F: forward; R: reverse

### Statistical analysis

The distribution of the genotypes and alleles as well as demographic characteristics (e.g., age, gender, smoking status, alcohol consumption and BMI) between CRC cases and healthy controls were analyzed by the χ^2^ test. The five SNPs were tested for Hardy-Weinberg equilibrium (HWE) analysis by using SHEsis-online (http://analysis.bio-x.cn/myAnalysis.php). Haplotype inference and linkage disequilibrium (LD) coefficients between alleles at different loci were computed by using SHEsis-online and Haploview 4.0. And then univariate logistic regression models were used to compare the distribution of SNPs between CRC cases and healthy controls. Multivariate logistic regression models were performed to compute adjusted odds ratios (AORs) and 95% confidence intervals (95% CIs). The variables age, gender, smoking status, alcohol consumption and BMI factors were selected as adjustment variables. Bonferroni correction for multiple testing was used to confirm statistical significance. All statistical tests in this study were two-sided and *P* < 0.05 was considered as statistically significant. All data were analyzed with the SPSS version 19.0 (SPSS China, Beijing, China) statistical package.

## Results

### Clinical characteristics of the subjects

The demographic characteristics of the population were shown in Table 2. The total number of CRC cases and healthy controls included in the study was 617. Among the subjects were 371 patients (238 males and 133 females, mean ± SD age 57.3 ± 12 years, range 20 to 83) and 246 healthy controls (139 males and 107 females, mean ± SD age 55.9 ± 13 years, range 20 to 82). The range of BMI was 15.4–32.7 (mean: 23.5) and 15.8–36.1 (mean: 23.6) in the CRC cases and healthy controls, respectively. Because the controls were frequency matched with the cases, there were no significant difference in the distribution of age, gender and BMI between the CRC cases and healthy controls (*P* > 0.05). Most of the patients had healthy lifestyle, and non-smoking and non-drinking patients accounted for 59.3% and 56.3%, respectively. There were no statistically significant difference between the groups with respect to smoking status (*P* = 0.305) and alcohol consumption (*P* = 0.588). Patients with colon and rectum cancer accounted for 32.9% and 67.1% of CRC respectively, and 43.4% of the patients had lymph node metastasis. Most of the patients were later Dukes stages.

**Table 2:** Demographic data of CRC cancer cases and healthy controls.

Demographic data	Cases no. (%)	Controls no. (%)	*P value*
All subjects	371 (100.0)	246 (100.0)	
Gender			0.056^a^
Male	238 (64.2)	139 (56.5)	
Female	133 (35.8)	107 (43.5)	
BMI, mean (range)	23.5 (15.4–32.7)	23.6 (15.8–36.1)	0.765^b^
Age, year			0.142^b^
Range	20–83	20–82	
Mean^c^	57.3 ± 12	55.9 ± 13	
≤ 40	35 (9.4)	26 (10.5)	
41–50	72 (19.4)	54 (22.0)	
51–60	106 (28.6)	70 (28.5)	
61–70	108 (29.1)	63 (25.6)	
≥ 70	50 (13.5)	33 (13.4)	
Smoking status			0.305^a^
Ever	151 (40.7)	90 (36.6)	
Never	220 (59.3)	156 (63.4)	
Alcohol consumption			0.588^a^
Ever	162 (43.7)	102 (41.5)	
Never	209 (56.3)	144 (58.5)	
Dukes stage			
A	50 (13.5)	—	
B	128 (34.5)	—	
C	118 (31.8)	—	
D	75 (20.2)	—	
Tumor site			
Colon	122 (32.9)	—	
Rectum	249 (67.1)	—	
Lymph node metastasis			
Yes	161 (43.4)	—	
No	210 (56.6)	—	

Body mass index (BMI).

^a^ Two-sided χ^2^ test for distributions between cases and controls.

^b^ Student’s *t* test.

^c^ Data are mean ± SD.

### Association between KRAS and VEGF 3’-UTR SNPs and susceptibility to CRC

Genotype distribution of one KRAS SNP and three VEGF SNPs were summarized in Table 3. Genotyping call rate of the candidate SNPs were 98.91–100%. The genotype distribution among the healthy controls were in HWE (*P* = 0.203 for rs1137188, *P* = 0.275 for rs10434, *P* = 0.743 for rs3025039 and *P* = 0.474 for rs3025053). At the VEGF rs3025039 locus, significant difference was found in genotype distribution of CRC cases and healthy controls (χ^2^ = 4.039, *P* = 0.043). When the rs3025039TT genotype was used as the reference, the C variant genotype was associated with an increased protection of CRC (AOR = 0.693, 95% CI = 0.485–0.989 for CC and CT+TT). Furthermore, the rs3025040 was in high LD with rs3025039 and had similar results, the CC variant genotype was also associated with an increased protection of CRC (AOR = 0.709, 95% CI = 0.524–0.963 for C/T and AOR = 0.693, 95% CI = 0.485–0.989 for CC and CT+TT) (data were not shown). However, these were no significant difference after Bonferroni correction. Other three SNPs (rs1137188GG, rs3025053AA and rs10434AA) were all not associated with CRC susceptibility when compared with their common genotypes.

**Table 3:** Statistical analysis of associations between the genotypes of SNPs and CRC risk.

Variants	Genotypes	Cases	Controls	χ^2^	*p*	p*	AOR	HWE
		(N = 371)	(N = 246)		Value^a^		(95% CI)^a^	*p* Value
KRAS rs1137188								0.203
	A	597 (80.5)	385 (78.3)	0.886	0.343	1.000	1.147 (0.864~1.524)	
	G	145 (19.5)	107 (21.7)					
	AA	239 (64.4)	146 (59.3)	1.621	0.182	0.728	1.258 (0.898~1.763)	
	AG + GG	132 (35.6)	100 (40.7)					
VEGF rs10434								0.275
	A	181 (24.4)	115 (23.4)	0.169	0.557	1.000	1.084 (0.828~1.420)	
	G	561 (75.6)	377 (76.6)					
	GG	209 (56.3)	142 (57.7)	0.116	0.580	1.000	0.911 (0.656~1.267)	
	AA + AG	162 (43.7)	104 (42.3)					
VEGF rs3025039								0.743
	C	604 (81.4)	422 (85.8)	4.033	0.049	0.196	0.728 (0.532~0.998)	
	T	138 (18.6)	70 (14.2)					
	CC	243 (65.5)	180 (73.2)	4.039	0.043	0.172	0.693 (0.485~0.989)	
	CT + TT	128 (34.5)	66 (26.8)					
VEGF rs3025053								0.474
	A	103 (13.9)	75 (15.2)	0.445	0.511	1.000	0.897 (0.649~1.240)	
	G	639 (86.1)	417 (84.8)					
	AA + AG	97 (26.1)	71 (28.9)	0.551	0.469	1.000	0.875 (0.609~1.257)	
	GG	274 (73.9)	175 (71.1)					

SNPs: Single nucleotide polymorphisms; HWE: Hardy-Weinberg equilibrium; OR: odds ratios; CI: confidence interval; AOR: adjusted odds ratios.

^a^ Adjusted for age, gender, smoking status, alcohol consumption and BMI using multivariate logistic regression.

* *P* value was corrected for Bonferroni correction.

### The association of KRAS and VEGF 3’-UTR SNPs and clinicopathological parameters of CRC

In genetic polymorphisms analysis, the association between KRAS and VEGF 3’-UTR SNPs and CRC risk was further analyzed by tumor site, tumor size, differentiation degree, invasive depth and TNM staging. As shown in Table 4, we found that the KRAS rs1137188 variant AA genotype was highly associated with tumor size (≥ 5 cm) (*P* = 0.01; Bonferroni-adjusted *P* = 0.04). The result revealed a significantly increased risk in CRC patients in the rs1137188 variant AA genotype when compared with that of the wild-type homozygous GG and heterozygous GA genotype. Meanwhile, the VEGF rs3025039 variant CC genotype was significant difference in tumor site (adjusted *P* = 0.024), tumor size (adjusted *P* = 0.019) and colorectal TNM staging (adjusted *P* = 0.032). The KRAS rs1137188 variant AA genotype was more evident in colon (adjusted *P* = 0.040). The VEGF rs3025053 variant GG genotype was more evident in colon (adjusted *P* = 0.030). However, these were no significant different after Bonferroni correction. At rs10434 locus, there were no difference in the risk estimates between the variant genotypes and CRC patients.

**Table 4:** The associations of SNPs polymorphisms and clinicopathological parameters of CRC.

Clinicopathological parameters	rs1137188		χ^2^	P*	rs10434		χ^2^	P*	rs3025039		χ^2^	P*	rs3025053		χ^2^	P*
	AA	GA+GG	(*p*^c^)		GG	AA+AG	(*p*^c^)		CC	CT+TT	(*p*^c^)		GG	AA+AG	(*p*^c^)	
Tumor site^a^																
Colon	88	34	4.715 (0.040)	0.160	72	50	0.532 (0.383)	1.000	90	32	5.504 (0.024)	0.096	99	23	5.007 (0.030)	0.120
Rectum	151	98			137	112			153	96			175	74		
Tumor size^b^																
≥ 5 cm	95	78	6.454 (0.010)	**0.040**	98	75	2.118 (0.153)	0.612	103	70	6.614 (0.019)	0.076	132	41	1.080 (0.245)	0.980
< 5 cm	79	34			74	39			84	29			80	33		
Differentiation^a^																
High	69	43	1.221 (0.511)	1.000	64	48	0.048 (0.938)	1.000	75	37	0.153 (0.939)	1.000	81	31	0.381 (0.788)	1.000
Medium	132	73			115	90			133	72			154	51		
Low	37	16			30	24			35	19			39	15		
Invasive depth^b^																
Serosa	57	43	0.951 (0.247)	0.988	54	46	0.2418 (0.139)	0.556	62	38	0.778 (0.527)	1.000	69	31	2.106 (0.194)	0.776
Tunica serosa	117	69			118	68			125	61			143	43		
TNM staging^b^																
Ⅰ + Ⅱ	94	67	0.931 (0.328)	1.000	96	65	0.040 (0.905)	1.000	96	65	5.395 (0.032)	0.128	116	45	0.828 (0.452)	1.000
Ⅲ + Ⅳ	80	45			76	49			91	34			96	29		

^a^ Statistical results for 371 patients Including colorectal cancer surgery and endoscopic examination patients data.

^b^Statistical results for 286 patients (Only including colorectal cancer surgery patients data, 85 endoscopic examination patients have no tumor size, invasive depth and TNM staging)

^c^ Adjusted for age, gender, smoking status, alcohol consumption and BMI using multivariate logistic regression.

* *P* value was corrected for Bonferroni correction.

## Discussion

We have investigated the important role of rs1137188 polymorphism of KRAS gene and rs3025039, rs3025040, rs3025053 and rs10434 polymorphisms of VEGF gene on the risk of the pathogensis, genetic sensitivity, and progression of CRC patients in a hospital-based case-control study. The study results had shown that CRC patients with KRAS rs1137188 AA genotype were more likely to have higher portion of tumor size (≥ 5 cm). The finding suggested that the rs1137188 may play an important role in the etiology of CRC. There were no significant association between the rest of polymorphisms and clinical outcome of CRC patients after Bonferroni correction.

The KRAS mutations, resulting in activation of the mitogen-activated protein kinase (MAPK) signaling pathway that mediates extracellular signals involved in cell proliferation, apoptosis and differentiation [22, 23]. The KRAS polymorphisms have been reported to influence the sensitivity and prognosis of various cancers of anti-targeted drug therapy, including lung cancer [24], gastric cancer [25], triple-negative breast cancer [26]. However, recent reports have mainly focused on the common genetic variations in the KRAS gene, published studies on the association between KRAS 3’-UTR SNPs and the susceptibility to various cancers are limited, Kim et al. [27] investigated KRAS rs9266 polymorphism was associated with risk of non-small cell lung and ovarian cancer. Kazmi et al [28] implicated that KRAS rs61764370 polymorphism was significantly associated with increased risk and prognosis of gallbladder cancer in North Indian population. Meanwhile, Dobre et al. [29] found that KRAS polymorphism was associated with statistically significant reduced survival of CRC. However, our study indicated that rs1137188 of KRAS gene in 3’-UTR was not associated with CRC susceptibility. Similar results have been observed in previous studies. Zhang et al. [30] investigated rs61764370 GT/GG polymorphism in KRAS 3’-UTR was not a genetic susceptible risk factor for CRC and could not be used as a biomarker for estimating cancer risk in Caucasian population. Langevin et al. [31] performed a review and meta-analysis to clarify the relationship between Let-7 microRNA-binding-site polymorphism in the 3’-UTR of KRAS and CRC outcome. Their results implicated that KRAS-LCS6 genotype was not associated with overall or progression-free survival (PFS) in the CRC patients. The reason of these results may be that the occurrence and development of CRC was related to a variety of factors, including gene-environmental interactions and ethnic ethics.

Another gene, VEGF, is a key player in the process of tumor angiogenesis and stimulates endothelial cell proliferation, survival and vascular maturation [32]. VEGF-related angiogenesis pathway is an important target of anti-cancer drug development [33]. There are a number of studies showing the influence of the VEGF SNPs on the risk and prognosis of various cancers, such as breast cancer [34] and non-small cell lung cancer [35]. However, few have reported the association of VEGF 3’-UTR SNPs with the susceptibility to CRC patients among Chinese. The association of polymorphisms in the VEGF pathway with CRC remains inconclusive. Rezaei et al. [36] found VEGF rs699947 polymorphism was correlated with development of breast cancer. Chen et al. [37] also reported that VEGF +936C/T gene polymorphism influenced the response to chemotherapy and overall survival of non-small cell lung cancer patients. Zhang et al. [38] found that VEGF rs3025039 was significantly associated with glioma susceptibility and might serve as genetic markers. Janardhan et al. [39] found that VEGF rs3025039 showed immense promise as a marker for disease aggression and recurrence and a factor for poor prognosis of epithelial ovarian cancer. Jeon et al. [40] carried out a case-control study to assess the relationship between VEGF 3’-UTR polymorphisms and CRC susceptibility in Koreans, and found that VEGF 1725G > A and 1451C > T may contribute to CRC susceptibility. In some solid tumors, expression of VEGF is significantly associated with angiogenesis and metastasis. The genetic polymorphisms in VEGF gene 3’-UTR can modify the potential binding sites of transcription factors, which lead to protein dysfunction and malformation. Lower VEGF expression can decrease tumor-related angiogenesis, growth and metastasis. However, our study pointed out that the SNPs in 3’-UTR of VEGF gene were not associated with CRC susceptibility in Han Chinese in Sichuan province after Bonferroni correction. Of course, the limited sample size and the differences of ethnicity could be the potential sources for inconsistent results.

There are several limitations in this study including the generalizability issue, since our study was restricted to the single Han Chinese patients. However, frequency of genetic polymorphisms often varies between ethnic groups. So further study is needed to clarify the generalizability of these findings to other ethnic populations. Moreover, the inadequate study design, such as a limited sample size, should be considered. The possible selection bias and information bias might have been present. The present study lacks information regarding lifestyle risk factors and clinical characteristics in the CRC patients, such as dietary intake, physical activity, aspirin use, PFS, 10-year PFS, overall survival and chemotherapy. These factors may also be associated with CRC risk. The mechanism by which polymorphisms in the KRAS gene and VEGF gene 3’-UTR affect development of CRC is still unclear. So, these results need to be confirmed further.

In summary, in this case-control study, our results indicated that KRAS rs1137188 AA may be a significantly increased risk for the Chinese CRC patients. However, the SNPs selected in our study were not adequate to support a role for CRC susceptibility, since after Bonferroni correction, the difference turns out to be insignificant. The study with a larger sample size are needed to draw firmer conclusions as to whether KRAS and VEGF SNPs in 3’-UTR are related to the risk of CRC. Further study of the relationship between KRAS and VEGF SNPs and survival analysis and the efficacy of chemotherapy-based drugs in CRC is needed. Additional functional studies will be done in order to better demonstrate the susceptibility and molecular mechanisms of CRC affected by KRAS and VEGF SNPs. However, our study identified clinical value of KRAS and VEGF 3’-UTR SNPs in Han Chinese in Sichuan province. It might provide a new measure for the individualized treatment of CRC patients in future.
